# 10-Year experience regarding the reliability and morbidity of radio guided lymph node biopsy in penile cancer patients and the associated radiation exposure of medical staff in this procedure

**DOI:** 10.1186/s12894-016-0166-2

**Published:** 2016-08-02

**Authors:** Ulf Lützen, Carsten Maik Naumann, Jens Dischinger, Marlies Marx, René Baumann, Yi Zhao, Michael Jüptner, Daniar Osmonov, Katrin Bothe, Klaus-Peter Jünemann, Maaz Zuhayra

**Affiliations:** 1 Department of Nuclear medicine, Molecular Imaging Diagnostics and Therapy, University Hospital Schleswig Holstein, Campus Kiel, Kiel, Germany; 2 Department of Urology and Pediatric Urology, University Hospital Schleswig Holstein, Campus Kiel, Kiel, Germany; 3 Northern German Seminar for Radiation Protection gGmbH at the Christian-Albrechts-University Kiel, Kiel, Germany; 4 Department of Radio Oncology, University Hospital Schleswig Holstein, Campus Kiel, Kiel, Germany

**Keywords:** Penile carcinoma, Sentinel lymph nodes, Lymph node staging, Tc 99 m-nanocolloid, Radiation exposure, Sentinel lymph node biopsy, SPECT/CT

## Abstract

**Background:**

The guidelines of the European Association of Urologists (EAU), of the German Society of Nuclear Medicine (DGN), and the European Society for Medical Oncology (ESMO) recommend sentinel lymph node biopsy (SLNB) for lymph node staging in penile cancer with non-palpable inguinal lymph nodes as one diagnostic method. Despite this, the method is neither widely nor regularly applied in Germany – the same applies to many other countries, which may be due to insecurity in dealing with open radioactive tracers. This study aims to assess the reliability and morbidity of this method, as well as the associated radioactive burden for clinical staff.

**Methods:**

Between 2006 and 2016, 34 patients with an invasive penile carcinoma and inconspicuous inguinal lymph node status underwent SLNB in 57 groins after application of a radiotracer (Tc-99 m nanocolloid). We collected the results prospectively. The reliability of the method was assessed by determining the false-negative rate. In addition, we evaluated complication rates and determined the radioactive burden for the clinical staff both pre- and intraoperatively.

**Results:**

SLNB was performed in 34 patients with penile cancer with non-palpable inguinal lymph nodes in 57 groins. In two patients inguinal lymph node metastases were detected by means of SLNB. In one patient recurrent inguinal lymph node disease was found after negative SLNB in both groins. Thus, the false negative rate was 3.13 % per patient (1/32 patients) and 3.51 % per groin (2/57 groins). The morbidity rate was 2.94 % per patient (1/34 patients) and 1.75 % per groin (1/57 groins). Radiation exposure for the clinical staff during this procedure was low at a maximum of ca. four μSV per intervention.

**Conclusions:**

SLNB is a reliable method with low morbidity that is associated with a low radiation burden for clinical staff. Due to the enhanced methodological and logistic demands, this intervention should be performed in specialized centres and in an interdisciplinary approach.

## Background

Application of Tc-99 m-labelled nanocolloid for pre- and intraoperative imaging of the sentinel lymph nodes (SLN) is a fully established standard method in malignant melanoma and breast cancer, both in Germany and internationally, and it is rooted firmly in the national and international guidelines of expert societies [1, 2].

For penile cancer, the European Association of Urologists (EAU), the German German Society of Nuclear Medicine (DGN), and the European Society for Medical Oncology (ESMO) also recommend sentinel lymph node biopsy (SLNB) in invasive primary tumours with a moderate degree of differentiation and non-palpable inguinal lymph nodes [3–5]. The former EAU categorization of the penile carcinoma as high-risk, intermediate-risk and low-risk is no longer in use [6].

In some few countries like the United Kingdom and the Netherlands, SLNB using open radioactive nuclides is an established and widely used procedure for penile cancer. In other countries like Germany, this procedure is neither regularly nor widely used. Despite the fact that urologists frequently deal with ionizing radiation from other sources, there is a lack of familiarity with the technique and the high methodological demands of SLN-procedures, therefor this could be one reason. A further cause could lie in former publications stating unreliability of the method of up to 15 % [7].

The aim of this prospective study is to establish both the reliability and the morbidity associated with SLNB after radio-labelling of sentinel lymph nodes with Tc-99 m nanocolloid. In addition, we aim to determine and assess the radiation burden for clinical staff resulting from the application of this method, and to compare it to similar procedures in other tumour entities.

## Methods

Being a university-based cancer center, we treat all types of penile cancer, as well as other tumour entities in an interdisciplinary team. Out of all patients who suffer from an invasive penile carcinoma and were assigned to our center by regional and national physicians, only those with non-palpable inguinal lymph nodes – this being the only pre-selection criterion – were included in this study in the period 2006 to 2016. During this period, 34 patients with an inconspicuous inguinal lymph node status in 57 groins were included in this study. 23 patients showed non-palpable inguinal lymph nodes bilaterally, 11 patients only unilaterally. The latter patients presented palpable inguinal lymph nodes in the contra lateral groin in the physical examination. All groins without palpable inguinal lymph nodes underwent SLNB with Tc-99-labelled nanocolloid, regardless of the palpation status in the contra lateral groin. The applied tracer was a pure gamma emitter with a half-life of six hours and energy of 141 keV.

All patients included in this study underwent initial preoperative physical screening, including palpation and additional ultrasound examination of the inguinal region. Preoperative cross-section images via magnetic resonance imaging (MRI) and computer tomography (CT) of the pelvic region, including the inguinal region, were not mandatory.

The median age of the patients was 63.5 (34–84) years. The details of the malignant disease, the tumour characteristics as well as the SLN diagnostic results and the results of the follow-up are presented in Table 1.

**Table 1:** Tumourstaging/-grading of patients with SLNB- and follow-up results

Tumourstaging/-grading	Patients (n)	Positive SLN	False-negative SLN
T1G1	2	0	1
T1G2	13	0	0
T1G3	4	1	0
T2G1	0	0	0
T2G2	9	1	0
T2G3	0	0	0
T3G1	2	0	0
T3G2	2	0	0
T3G3	2	0	0

**Fig. 1:**
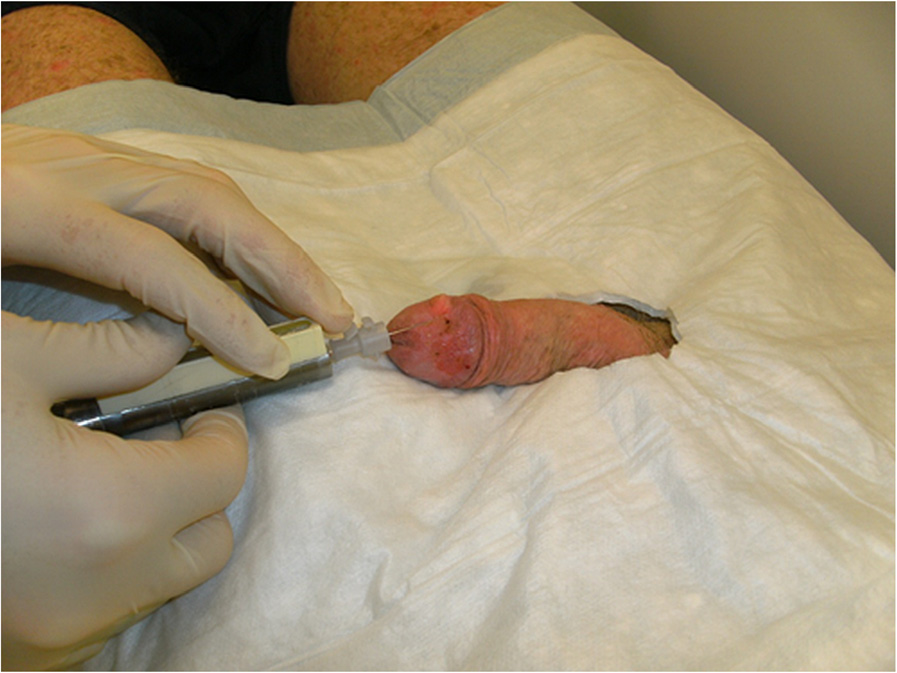
Pre-operative peritumoural intracutaneous injection of the radioactive tracer (Tc 99 m-labelled nanocolloid)

**Fig. 2:**
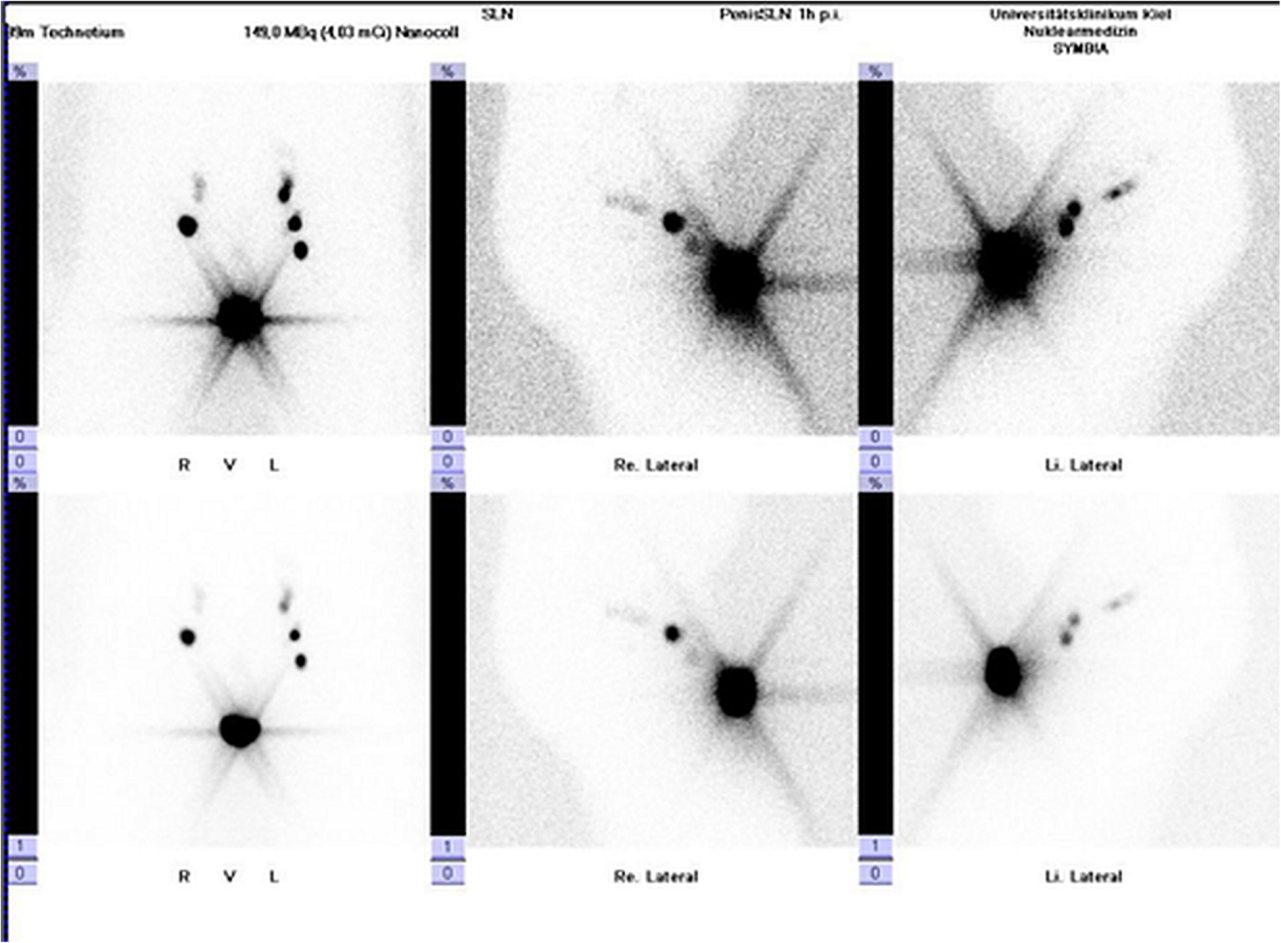
Pre-operative scans of Sentinel-lymph nodes by means of planar scintigraphy in several projections and indirect body contouring via a Co 57-planar source: Evidence of so-called “hot spots” in the inguinal region bilaterally (3 SLN left, 2 SLN right) as well as in the region of the tumour

Nuclear medical imaging of SLN was done following a-two-day protocol [8]. On the preoperative day, the patients received an intradermal peritumoural injection of the radio tracer under local anesthesia (Fig. 1). We applied an overall activity of 150 MBq, Tc-99 m-labelled nanocolloid. We performed lymphatic drainage scintigraphies in several projections (at least four): at the earliest on the injection day at least one hour p. i., − or, in the case of lacking or retarded lymphatic drainage, on the morning before the surgical intervention. For indirect body contouring, the emission measurements for generation of planar images were performed additionally by means of a Co-57 planar source (Fig. 2). Image acquisition was done with a twin head gamma camera (Siemens, ECAM and Symbia T).

**Fig. 3:**
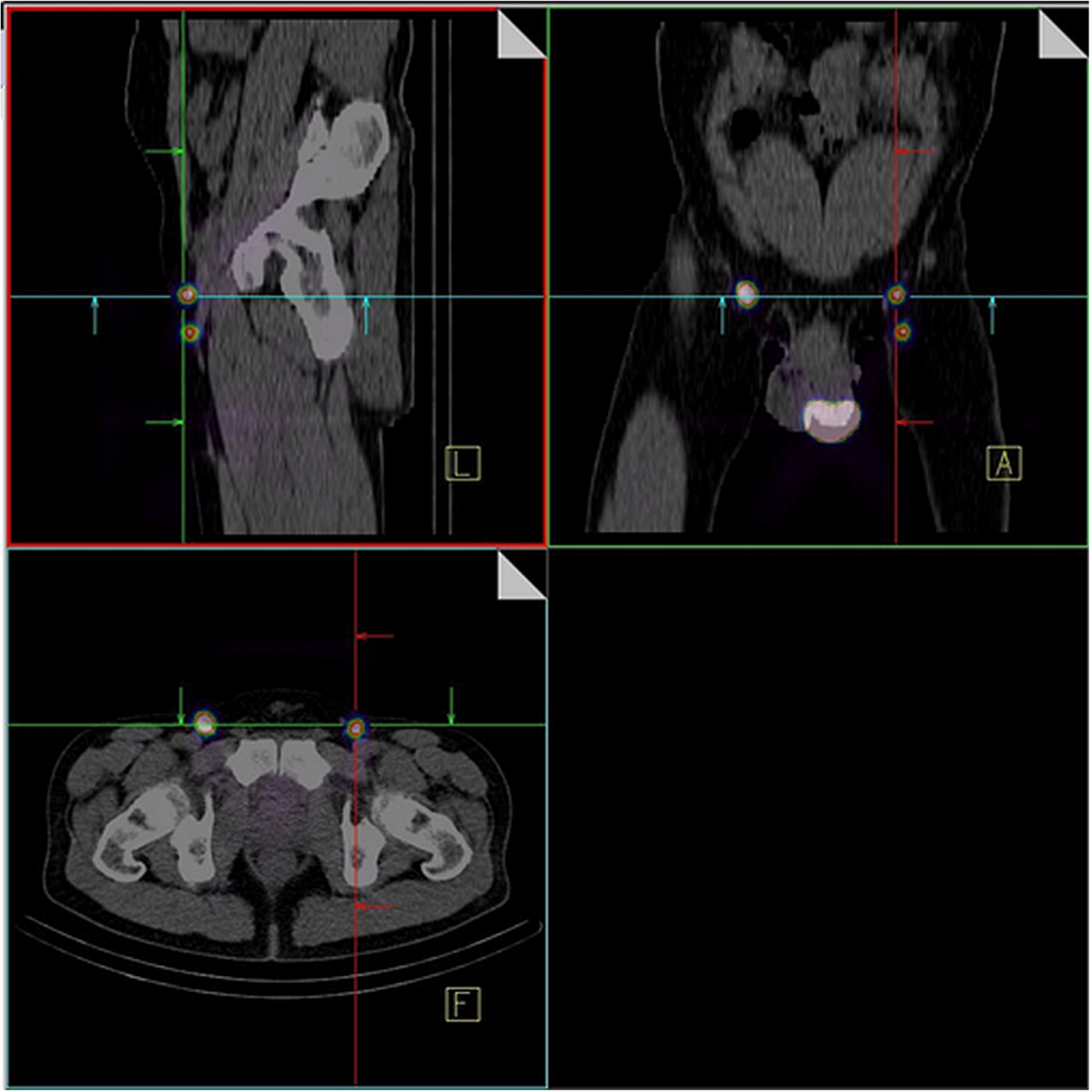
Pre-operative scans of sentinel lymph nodes by means of SPECT/CT: Evidence of so-called “hot spots” in the inguinal region bilaterally (2 SLN left, 1 SLN right) as well as in the region of the tumour

In addition we performed single-photon-emission computed tomography/computed tomography (SPECT/CT) of the lower abdomen, the pelvic abdomen and the inguinal region with a twin head SPECT/CT-hybrid camera (Siemens, Symbia T and Symbia Intevo). CT-data were acquired in a so-called low-dose technique. The scans of the functional imaging were fused with the co-registered CT-scans (Fig. 3). In this context, morphological imaging enabled attenuation correction of the emission data on the one hand, as well as easier anatomical allocation of the radio-marked lymph nodes, thus facilitating better surgical planning, on the other.

Subsequent to the imaging process, we identified and marked the SLN by means of a Co-57 pen as well as a felt-tip pen on the skin surface. In addition we performed a perilesional intradermal injection of patent blue immediately before the surgical intervention. Intraoperatively, the SLN were identified both visually, via the blue hue accrued in the lymph nodes, as well as by measuring the radioactivity by means of a gamma probe. In the case of preoperative non-visualization of radioactive labeled lymph nodes in scintigraphy the day before surgery, an intraoperative exploration, augmented by the gamma probe, was done the following day. All detectable radioactive-labeled, blue stained as well as clinically suspicious lymph nodes that were not detected by preoperative physical examination (e.g. due to obesity) were removed as recommended [8].

Each enrolled patient of this study received a prophylactic antibiotic therapy as a single shot application of cephalosporin prior to surgery. Incisions of approximately four cm were made for extirpation of the lymph nodes, following the relaxed skin tension lines. All patients were provided with drains at the end of surgery. The drains were left until the flow-rate was equal or smaller than 20 ml per 24 h. The mean respectively median operation time was 130.3 min respectively 120 min. On average, 3.2 lymph nodes (median 2/range 1–11) per patient were resected. Neither compression bondages nor stockings were applied. The mean, respectively median length of the postoperative hospitalization was 5.6, respectively 5.0 days. Histopathological examination of the entire SLNs was done in 100 micrometer (μm) sections under additional hematoxylin-eosin staining. In case there was evidence of positive SLNs with malignant tumour cells, we performed a radical lymphadenectomy (LAE) in a two-stage surgical intervention in the affected inguinal region and if necessary a systemic cytotoxic therapy. In our cohort, inguinal LAE was necessary in two cases. In one case, chronic lymphedema of the lower extremity resulted. In the other case, there were no unexpected events in terms of morbidity.

Three different urologists performed the SLNB-procedures. Preoperatively, also three different nuclear medicine physicians performed the applications of the radioactive tracer. Morbidity and disease-free survival (DFS) after SLNB was evaluated during clinical out-patient follow-up examinations.

As a reference standard, a negative groin was defined as a histologically negative SLN status and uneventful follow-up without nodal recurrence. A positive groin was defined as a histologically positive SLN status or lymph node recurrence during the follow-up period. Based on this definition, we compared SLNB to the reference standard of follow-up examinations. Follow-up was done in the same way in all histologically negative SLN-patients i.e. by physical examination, including inguinal node palpation of the groins in three-monthly intervals for the first two years, every four months in year three and in six-monthly intervals in the fourth and fifth year after diagnosis.

For assessment of the radiation exposure of the involved medical staff in this procedure, we performed both pre- and intraoperative measurements. To this end, we applied a dose rate meter (Berthold, LB123 and Berthold, TOL F) to measure the dose rate (DR) and the time needed for each procedural step. Using these two measured parameters we calculated the effective dose and the hand dose of the involved staff. As a control we used an additional digital personal dosimeter (Polimaster, PM1621MA) for determination of the effective dose of the surgeon. During the measurements, the digital personal dosimeter was placed in the breast pocket of the surgeons’ shirt. Prior to radiation measurements during the SLN-procedures, the background radiation in all rooms was determined and subtracted. In view of the fact that the lower detection limit of the thermoluminescence ring dosimeter (TLD) of fingers is >100 μSv, we abstained from using TLD finger rings during pre- and intraoperative measurements. TLD is only of limited use due to the low applied activity and the required pre- and intraoperative times during the procedure, and it is inferior to measurement via sensitive dose rate meters. The assessment comprises the following: preoperatively, the identification of dose values for the staff from nuclear medicine, and intraoperatively, that for anesthetists, surgical staff and the surgeon, taking into account the required time span, the distance to the radiation source, and the intensity of applied activity.

Prior to the procedure, all patients were duly informed about the details of the measures and gave their written informed consent for this guideline-conform procedure. In addition, the study was approved by the Ethics Committee of Kiel University (AK D 426/07).

## Results

### Reliability and morbidity

In two patients, metastatic disease in the SLN was proven by means of SLNB with subsequent histological examination (Table 1). There were no false-positive results. In one patient, bilateral recurrence of inguinal lymph node metastases was diagnosed four months after histologically negative SLNB. After clinical suspicion of the inguinal lymph node recurrence a bilateral inguinal LAE was conducted. The mean resp. median disease-free survival of patients with a negative SLN result was 52.26 resp. 51 (4–109) months. One patient died from the consequences of a stroke ca. five months after the intervention. A further patient died from the consequences of a renal cell carcinoma ca. twelve months after treatment of penile carcinoma. All other patients were examined as part of the regular follow-up.

The current data, which covers a period of ten years, shows a false-negative rate of 3.13 % in relation to the number of patients (1/32 patients) and 3.51 % in relation to number of investigated groins (2/57 groins). Assessment of morbidity after SLNB alone showed one case of a prolonged inguinal lymphorrhea, which disappeared spontaneously by leaving the intraoperatively placed drain. SLNB-associated morbidity was thus calculated as 2.94 % per patient (1/34 patients) and 1.75 % per groin (1/57 groins).

The patient who had a false-negative SLNB result during follow-up had a rather unusual clinical course and deserves special attention. In this patient, SLNB was performed during a secondary resection, i. e. after resection of the primary tumour ex domo with an unclear resection status. Despite a unilaterally unsuccessful visualization of a radio-labelled SLN, we performed follow-up investigations on the basis of the T1 stadium diagnosed ex domo and the G1 differentiation as well as a R0 resection according to the guidelines. Unfortunately in this case tumour specimens were not available for our pathologists for re-evaluation.

During a sonographic follow-up examination four months after surgery, we found a bilateral lymph node recurrence, which was successfully resolved by surgical intervention as mentioned above. The patient has been disease-free during further follow-up.

Based on cross-counting (Table 2) and chi-square test we calculated for SLNB a sensitivity of 66.67 % and a specificity of 100.00 %, with a 95 % confidence interval (CI) of 9.5 to 99.2 % (sensitivity) respectively of 88.8 to 100.0 % (specificity).

**Table 2:** Cross-table of the results of SLNB and follow-up and of SLNB alone

		Results of SLNB and follow-up (n)		
		Histo. positive	Histo. negative	Sum
Results of SLNB alone (n)	histo. positive	true positive 2	false positive 0	all positive 2
	histo. negative	false negative 1	true negative 31	all negative 32
	sum	all true findings 3	all false findings 31	all findings 34

### Radiation exposure of involved staff

Table 3 shows the pre- and intraoperatively measured mean DR of the applied radioactivity at various distances to the radiation source.

**Table 3:** Pre- and intraoperative mean dose rates during SLN procedure (2-day protocol) with a 150 MBq Tc-99 m nanocolloid at various distances to the radiation source/patient

Distance	Mean dose rate (μSv/h)
Preoperatively	
Directly on the injection needle (0 cm)	200
Hands of nuclear medicine physician (10 cm)	100
Position of nuclear medicine physician (30 cm)	10
Position assisting staff (50 cm)	2
Intraoperatively (prior to resection of the tumour)	
Hands of surgeon (10 cm)	20
Surgeon (30 cm)	2.2
Surgical staff/physician (50 cm)	0.5
Anesthetist (200 cm)	0.13
Intraoperatively (after resection of the tumour)	
Directly on the sample (0 cm)	15
Hands of surgeon (10 cm)	1.5
Surgeon (30 cm)	1.1
Surgical staff/physician (50 cm)	0.3

In consideration of the measured exposure time of 45 min with a mean distance of 30 cm to the patient prior to resection of the primary tumour, and a exposure time of 120 min after resection of the primary tumour, we calculated a mean effective dose for the surgeon of ca. four μSv per patient (2.2 μSv/h × 0.75 h + 1.1 μSv/h × 2 h), based on the measured mean dose rate values presented in Table 2. The mean effective dose for the surgeon as controlled by the digital dosimeter is ca. 4.06 μSv for the entire surgical intervention. The values thus concur closely with the estimated values.

The mean hand dose of the surgeon is calculated to be ca. 18 μSv per patient (20 μSv/h × 0.75 h + 1.5 μSv/h × 2 h).

The surgical staff can keep a distance of at least 50 cm to the patient resp. the surgical area during the entire procedure. In the worst case, this can result in a mean effective dose of 0.98 μSv per patient (0.5 μSv/h × 0.75 h + 0.3 μSv/h × 2 h) for these persons. The hand dose is negligible here.

Table 4 shows a summary of the mean pre- and intraoperative radiation exposure for the clinical staff as has been measured or estimated above for penile carcinoma SLNB, in comparison with the mean dose values that apply in malignant melanoma or breast cancer SLNB procedures.

**Table 4:** Pre- and intraoperative mean radiation exposure of the clinical staff by SLNB in different tumour entities

	Tumour entity					
	Penile carcinoma		Malignant melanoma		Breast cancer	
applied activity [MBq] Tc-99 m-labelled nanocolloid	150 2-day protocol		45 1-day protocol		150 2-day protocol	
	mean Effective Dose E [μSv]	mean Organ-dose of the hands H_hands_ [μSv]	mean Effective Dose E [μSv]	mean Organ-dose of the hands H_hands_ [μSv]	mean Effective Dose E [μSv]	mean Organ-dose of the hands H_hands_ [μSv]
clinical staff						
nuclear medicine physician	105	20	2	10	0.6	10
nuclear medicine technologist	0.5	10	0.5	10	0.2	10
surgeon	4.0	100	0.5	7	0.6	5
anesthetist	1.0	-	0.7	-	0.4	-
surgical staff	1.0	-	0.5	-	0.4	-

## Discussion

### Reliability and morbidity

The indication for an LAE in penile carcinomas has been under controversial discussion for many years, especially with respect to clinically and sonographically inconspicuous inguinal lymph nodes [9]. The degree of metastatic spread and the respective appropriate therapeutic management have a significant impact on patient survival. Classic inguinal LAE is associated with a substantial morbidity rate of up to 87.5 % according to the literature (e. g. wound healing problems, generation of seroma or lymphorrhea, etc.) [9, 10].

Only 20 to 25 % of patients with clinically negative inguinal lymph node status harbour occult metastases. By reverse logic, this means that ca. 75 to 80 % of patients with clinically inconspicuous inguinal lymph nodes undergo the increased of morbidity risk going through this surgical intervention in vain. A tentative watchful waiting approach, however, involves a high risk of tumour-related mortality. Results from the literature show that resection of metachronous, clinically evident metastases during follow-up procedures significantly diminishes the long-term survival chances of these patients, compared to immediate resection of occult metastases, from 84 to 35 % [9, 11]. Thus we did not choose the tentative approach in the patients included in this study.

Alternative, non-invasive functional and morphological imaging procedures such as positron-emission tomography/computed tomography (PET/CT) or MRI and CT alone do not have sufficient sensitivity in patients with clinically inconspicuous inguinal lymph node status [12–15]; they are not seen as useful and have thus not been used as obligatory imaging tools in this study.

EAU guidelines recommend performance of SLNB in penile carcinomas with a tumour stadium ≥ T1G2 and non-palpable inguinal lymph nodes (cN0) [6]. If SLN-labelling with radioactive tracers is not available, the guidelines alternatively recommend a fine needle aspiration biopsy (FNAB) of the inguinal lymph nodes with subsequent cytopathological examination.

Regarding FNAB, however, the low sensitivity of only 39 % in penile cancer has to be taken into consideration [16]. A further alternative is to base the treatment strategy on individual risk factors [6]. A decision in favor or against inguinal SLNB based on risk factors like tumour stage and/or tumour differentiation for metastatic risk assessment involves substantial uncertainties. All patients with proven or clinically evident invasive penile cancer without palpable inguinal lymph nodes underwent SLNB. In case of uncertainty concerning the invasiveness of the tumor, we performed preoperative radioactive lymph node labeling including scintigraphy. SLNB was only performed if rapid frozen section during surgery showed at least a T1-carcinoma. However, the reliability of histopathological tumour grading based solely on rapid frozen section is unclear and has not been described in literature yet. Even paraffin-embedded specimens show a high degree of inter-observer variability among pathologists with respect to tumour staging and tissue differentiation [17, 18]. Taking these facts into account, we accepted a possible over diagnosis/-treatment in a very low number of patients with T1G1-tumours by SLNB. As mentioned before, the remarkable thing was that the only relapse due to a false-negative SLNB-procedure was proven in a patient with a T1G1-disease, who had undergone primary surgery ex domo. For this reason, we performed SLNB in each invasive carcinoma of this entity.

The results from our study show that SLNB after radio-labelling with Tc 99 m-nanocolloid is a reliable method for lymph node-diagnostics.

The false-negative rate of 3.13 % in relation to the number of patients (1/32 patients) and 3.51 % in relation to number of investigated groins (2/57 groins) is comparable to that of the results of the Dutch workgroup around Leitje et al. [8], but significantly better than the results of the Swedish workgroup around Kirrander et al. [7], whose false-negative rate was 15 % in a study published in 2012. In the aforementioned Dutch study the false-negative rate was seven percent [8]. In the study by Leitje et al. the results are based on the number of the examined inguinal regions and have not been calculated per patient as in our study. The Dutch study observes that the metastases rate of ten percent is comparatively low, which implies the question whether the high logistic and methodic demands of SLNB with radioactive nuclides can be justified.

Compared to other studies e.g. Lam et al. [19] or Kroon et al. [20] who report rates of 28 respectively 22 % of histologically positive lymph nodes under employment of SLNB, our rate of metastatic disease is lower. The reason might be that the patients enrolled in the study of Lam et al. had a significantly higher risk of metastatic disease, as can be seen in the lower number of T1-tumours (42 %) compared to our study (56 %). More importantly, the study of Lam et al. included a higher rate of G3-tumours (53 %). In our study the G3-tumour rate is only 18 %. Moreover, the study by Kroon et al. included only patients with T2- and T3-tumours, while T1-tumours were not taken into account, in contrast to our study. These facts and the criteria leading to the low number of primarily positive lymph nodes using the SLNB-procedure might be responsible for our statistical results of sensitivity, specificity, and the 95 % CI. Although our study includes a large patient cohort compared to other German studies, the number is rather small by international standards [8]. It is well possible that higher rates of metastases are found in larger patient cohorts in the future.

The following consideration shows the clinical implication of radio-labelled SLNB: in the current study, we were able to prove a negative nodal status by SLNB with sufficiently long follow-up periods in 31 patients.

Based on the now obsolete risk classification stated in the 2004 EAU-guidelines [21], 19 high-risk patients would have undergone obligatory LAE and further 13 intermediate-risk patients would have been candidates for a facultative classic inguinal LAE without any further diagnostic gain. In this context it has to be mentioned that the patient who had a bilateral inguinal lymph node recurrence after negative SLNB was a low-risk patient (T1G1/Table 1) and was diagnosed and underwent R0 resection ex domo where SLNB was not indicated. Unfortunately, tumor specimens were not available for re-evaluation by our pathologist.

Based only on the SLNB result, we were able to spare these patients the severe morbidity associated with classic inguinal LAE while guarding diagnostic safety. As a consequence we were able to reduce morbidity in our patient cohort to 2.94 % per patient (1/34 patients) and 1.75 % per groin (1/57 groins) by using SLNB.

In view of the results of our study, especially the case of the patient who underwent ex domo surgery of the primary tumour, the question arises whether SLNB after application of radioactive lymphogenic tracers can also be safely applied as a secondary intervention, i.e. after resection of the primary tumour.

Graafland et al. [22] performed a metachronous SLNB in 40 patients after earlier resection of the primary tumour. The reported results of a two-stage procedure are comparable to those of primary SLNB.

Our results cannot be used to confirm the findings by Graafland et al. as we encountered lymph node recurrence after a false-negative SLN-procedure four months after secondary SLNB during follow-up. In the currently described patient collective after primary SLNB the false-negative rate is zero percent.

### Risks and benefits of SLNB and classic inguinal LAE

As mentioned the morbidity of a classic inguinal LAE is as high as 87.5 % [9, 10]. Due to the fact that lymph node metastases can only be proven in ca. 20 to 25 % of patients with clinically non-palpable inguinal lymph nodes [3], this treatment later reveals itself to have been unnecessary in 75 to 80 % of these patients.

As already stated, the Dutch workgroup around Horenblas, like some other workgroups, reported false negative rates of approximately 15 % during the early implementation phase of the SLNB-procedure [7, 23]. Data from an analysis by Neto et al. require a critical view [24]. These comprise studies examining the results of SLNB including patients with palpable inguinal lymph nodes. SLNB is not recommended by the expert society guidelines in patients with palpable inguinal lymph nodes due to its high unreliability [3]. In our study, we could show excellent reliability of SLNB, with a false negative rate of only 3.51 % (2/57) per groin respectively 3.13 % (1/32) per patient. Like us, Lam and coworkers report a false negative rate of 5 % (in relation to the groins) respectively 6 % (in relation to the patients) in a cohort of 264 patients. The faulty procedures occurred mainly during the implementation of the method [19]. Concerning the false negative rates of classic inguinal LAE for staging, no data in the literature are available yet.

One of the limitations of SLNB might be that tumour cells can lead to obstruction or occlusion of the lymphatic drainage pathways and can either cause a complete blockage or a rerouting of the radio tracer [23, 25]. This aspect can lead to restraints of SLNB. The risk of a relevant modification of lymphatic and thus tracer drainage depends on the metastatic load in the lymphatic pathways and in the lymph nodes. The risk of lymph node metastases in patients with palpable lymph nodes is about 50 % and therefore much higher than in those with an inconspicuous inguinal lymph node status during palpation of the groins (20 to 25 %). For this reason, expert society guidelines do not recommend SLNB in patients with palpable inguinal lymph nodes, as has been mentioned before [3].

The validity of clinical groin palpation within the physical examination is influenced by the physical constitution of the patient on one hand, and by the experience of the examiner on the other. In our study, one patient developed a bilateral lymph node recurrence four months after histologically negative SLNB. The assessment of the inguinal lymph node status was impeded by corpulence. Obesity is capable of veiling otherwise palpable and potentially tumour-infested lymph nodes, thus wrongly leading to the conclusion that the patient is eligible for SLNB, which presents another limitation of this staging procedure. This risk of misjudgment could be minimized by preoperative sonography of the groins, as a supplement to clinical palpation.

As mentioned, a further possible source of errors in the application of this procedure is associated with the two-stage approach of SLNB after surgical removal of primary tumour. While Graafland et al. [22] report uniform results in 40 cases of a metachronously performed SLNB, we believe that surgery-related modifications of the tracer drainage, e.g. through scar formation or edema, might be a reason for the false-negative SLNB in one patient of this study.

#### Radiation exposure of the involved staff

German radiation protection laws prescribe that any indication for the application of ionizing radiation, also that of open radioactive nuclides, is mandatorily bound to a medical indication identified by a physician with the necessary expertise [26]. Thus the resulting benefit of this procedure for the patient outweighs the potential risk of ionizing radiation.

Based on the specifications of the German radiation protection commission (Strahlenschutzkommission, SSK), we have calculated an effective dose of 0.5 to 1.5 Millisievert (mSv) in patients undergoing the SLN procedure according to a so-called two-day protocol under application of the above-described tracer with an activity of 150 MBq [27]. Our dosimetric calculations fit to the data from the literature. Application of an additional low-dose CT in the pelvic region leads to an additional effective dose of 1–2 mSv [27]. We were able to confirm this by our calculations (CT Expo Version 2.4/2015/G. Stamm, H. D. Nagel).

According to our measurements and calculations, the effective dose for the nuclear-medical staff of 0.5 μSv (nuclear medicine technologists) resp. 1.5 μSv (nuclear medicine physicians) is relatively low, which is owed to the fact that preoperative application of the tracer, despite its high radioactive load of 150 MBq, requires little time, and thus makes the exposure time much shorter than the intraoperative exposure. The effective dose values for the nuclear medical staff are comparable to those occurring during application of this kind of procedure in other tumour entities like breast cancer or malignant melanoma.

Compared to other procedures in nuclear medicine, sentinel lymph node diagnostics with radioactive tracers is associated with a relatively low radiation burden for the nuclear medical staff.

Physicians and technologists in nuclear medicine are allowed to be exposed to radiation by profession and are as such allocated to at least Category B with a maximum effective dosage of 6 mSv (6000 μSv)/a and a maximum hand dosage of 150 mSv (150000 μSv)/a. If the nuclear medicine physician was to only perform this kind of procedure, up to 4000 procedures per year would be eligible. If the nuclear medicine expert was allocated to category A of professional radiation exposure, maximum eligible effective dose would even be 20 mSv/a with a maximum hand dose of 500 mSv/a [26].

In this tumour entity, organ-preserving surgical resection of the primary tumour can be technically demanding and time-consuming. Thus the surgeon is the person with the highest effective dose exposure as shown by our pre- and intraoperative measurements (maximum of ca. four μSv per patient).

Compared to other tumour entities, the effective dose for the surgeon of penile carcinoma is higher (Table 3). This is due to the fact that more time is required in penile cancer surgery, − i.e. for the preparation of the primary tumour and subsequent functional reconstruction of the urethra -, compared to resection of malignant melanoma in unproblematic localizations or mastocarcinoma resection including the SLN; this considered, the radiation exposure time in penile cancer is longer.

Surgical staff including urologists is not classified as “radiation-exposed by profession” and is eligible for exposure up to a maximum effective dose of 1 mSv (1000 μSv)/a and a maximum hand dose of 50 mSv (50000 μSv)/a [26]. The effective dose for surgical staff measured by us amounts to ca. 1.0 μSv per patient (Table 3). Based on these results, this means that the surgical staff could perform ca. 1000 SLN interventions per year without exceeding the threshold value of German radiation protection regulations, while the surgeon could perform ca. 250 interventions per year [26, 28] In addition, it has to be taken into account that penile carcinomas both in Europe and in the U.S. are much more rare than for example breast cancer or malignant melanoma [29]. While the effective dose for the surgeon per patient is thus higher than in the other mentioned entities, the overall dose remains relatively low and far below the threshold of the radiation protection laws.

Our calculations and measurements as well as the data in the literature on staff radiation exposure [27] have shown that both the staff in nuclear medicine and the surgical staff can be expected to undergo only very limited radiation exposure, which is well within the legal boundaries. A tentative attitude towards SLNB with radioactive lymph node labelling cannot be justified on the basis of radiation protection aspects. Worries and fears on this score are unfounded. For more transparency, surgeons in Germany who perform SLNB with radioactive labelling have been obliged since 2011 to complete a 6 h–training in radiation protection [28]. Regardless of this, the procedure has high methodical demands and requires an experienced and interdisciplinary team consisting of a specialist in nuclear medicine, an urologist and a pathologist. This method could be facilitated through multidisciplinary centres or by collaborative clinical setups.

## Conclusions

SLNB with SLN-labelling via Tc-99 m nanocolloids in penile cancer is a valuable diagnostic method associated with low morbidity rates. It offers a high degree of reliability, especially when performed during surgical resection of the primary tumour. The method has high methodological and logistical demands as it among other things requires an interdisciplinary team. In due consideration of radiation protection laws, the resulting radiation exposure for clinical staff during the SLN- procedure is low, and a tentative attitude towards this approach is not justified.
